# Practices Supporting Electronic Health Record Transitions: Lessons from Four US Healthcare Systems

**DOI:** 10.1007/s11606-023-08279-0

**Published:** 2023-10-05

**Authors:** Seppo T. Rinne, Julian Brunner, David C. Mohr, Adena-Cohen Bearak, Ekaterina Anderson

**Affiliations:** 1 Center for Healthcare Organization and Implementation Research (CHOIR), VA Bedford Healthcare System, Bedford, MA USA; 2 Pulmonary & Critical Care Medicine, School of Medicine, Boston University, Boston, MA USA; 3 Center for the Study of Healthcare Innovation, Implementation & Policy, VA Greater Los Angeles Health Care System, Los Angeles, CA USA; 4 Center for Healthcare Organization and Implementation Research, VA Boston Healthcare System, Boston, MA USA; 5 Boston University School of Public Health, Boston, MA USA; 6 Department of Population and Quantitative Health Sciences, University of Massachusetts Medical School, Worcester, MA USA

## Abstract

**Background:**

Electronic health record (EHR) transitions are common and complex organizational changes, yet limited published literature is available to guide health systems that are changing from one EHR to another. Clinicians and staff end users at sites that have undergone EHR transitions may have critical insights that could inform future transitions.

**Objective:**

To assess end user perspectives on organizational practices that support successful EHR transitions.

**Design:**

Multi-site qualitative study of end users at healthcare systems that transitioned to a new EHR (either Epic or Cerner) within the prior 3 years.

**Participants:**

Forty-two participants, including providers, clinical leaders, and informaticists at four geographically and organizationally diverse US healthcare systems.

**Approach:**

We conducted semi-structured telephone interviews, which were audio-recorded and transcribed. We used content analysis to identify key practices that influenced EHR transition success.

**Key Results:**

Participants described specific organizational practices that they found most helpful in supporting EHR transitions, and these practices transcended individual sites and EHR systems. We categorized practices based on how they were described relative to the stage of implementation. During pre-go-live, recommended practices included communicate rationale and anticipated outcomes of the EHR change; understand baseline workflows; and plan for appropriate customization. During go-live, recommended practices included personalize training and support; invest in robust internal support; reduce workload expectations; and proactively address challenges. The recommended post-go-live practice was to continue to invest in the change.

**Conclusions:**

Our findings may act as a roadmap for future EHR transitions by identifying specific and actionable organizational practices across stages of implementation. These recommendations highlight the role of health system leaders in preparing for the organizational change, working with and supporting end users, and addressing challenges that arise.

**Supplementary Information:**

The online version contains supplementary material available at 10.1007/s11606-023-08279-0.

## INTRODUCTION

Transitioning from one electronic health record (EHR) to another is a complex organizational change that impacts every facet of care delivery. These transitions are not just technology changes but are rooted in sociotechnical and culture change.^1^ Successful EHR transitions must use organizational practices that engage clinicians and help them navigate these complex changes. Yet, limited guidance is available. Lessons from studies of initial paper-to-EHR implementations^2–5^ may not directly apply to transitions that must simultaneously de-implement old EHRs and implement new ones.^6^ Single-site EHR transition studies exist^7,8^, but these studies are not able to assess generalizable strategies that transcend site-specific contexts. In the absence of these data, several authors have developed pragmatic recommendations to guide healthcare organization undergoing EHR transitions.^9–13^ Additional research is needed to assess how these recommendations align with end user experiences during EHR transitions.

Clinicians and staff at sites that have undergone EHR transitions have critical insights that could inform future transitions. EHR end users are the primary target of change management efforts, and they may have valuable information on the challenges they experienced as well as the best practices that supported them during the change. Despite the importance of clinician voice, limited research has explored their perspectives on EHR transitions.

The Department of Veterans Affairs (VA) is currently transitioning from its long-standing homegrown EHR to a new EHR by the Oracle Cerner corporation.^14^ This massive organizational change is the largest EHR transition globally and has already experienced notable challenges.^15–18^ Our primary research objective was to explore end user perspectives on organizational practices that facilitate successful EHR transition. These lessons could provide guidance to VA and other healthcare systems undergoing EHR modernization efforts.

## METHODS

We conducted a multi-site qualitative study of four healthcare systems that transitioned to new EHR systems in the prior three years. We chose sites that had transitioned to Epic or Cerner EHRs (two sites each) because they currently hold the largest EHR market share (32.9% and 24.4% of share of US Hospital EHR market, respectively),^19^ and for relevance to VA’s planned transition to the Oracle Cerner EHR system. The study was approved by the VA Bedford Healthcare System Institutional Review Board.

We selected sites based on our personal knowledge of their EHR transition, information from vendor websites, and media reports. We intentionally selected sites that were geographically and organizationally diverse. From each site, we identified a convenience sample of potential participants using healthcare systems’ websites, professional societies, personal contacts, and snowball sampling. Identified individuals were invited to participate by email.

Two trained qualitative researchers (EA, JB) conducted semi-structured interviews between September 2019 and July 2020. Interviews lasted approximately 50 min (range: 29–86 min). Participants were compensated with a $100 gift card. The interview guide (see Appendix 1) was designed to address general experiences with the EHR transition, perceptions of change management practices, and perceived individual and organizational impacts of the transition. Interviews were audio-recorded and professionally transcribed.

We applied qualitative content analysis procedures to analyze the interviews.^20^ As a team, we developed a codebook that included a priori concepts from existing literature on EHR transitions. Emergent concepts were identified from the interviews. Qualitative data analysis software ATLAS.ti version 8 was used for coding.^21^ First, all authors independently coded three transcripts and discussed their experiences to align coding practices and refine the codebook. Subsequently, each transcript was coded by a single author and their work was reviewed by another author. Coding disagreements were resolved via team discussion. After coding was completed, first author (SR) reviewed the coded data across and within major categories of interest (e.g., training, planning, communication) to generate summaries of participants’ perceptions of practices that facilitate and/or hamper successful EHR transitions. We include representative quotes with bolded font to emphasize key segments.

## RESULTS

Across the four institutions, we interviewed 42 participants, of which 37 were providers (e.g., medical doctors and nurse practitioners). Other participants included local informaticists and EHR trainers (Table 1).

**Table 1:** Interview Participants and Site Characteristics

	Site A	Site B	Site C	Site D
Interview participants	13	9	12	8
Institution type	Integrated delivery system	Community hospital system	Academic medical center	Integrated delivery system
Geographic region	Multi-region	South	Northeast	Multi-region
Approximate number of employees	59,000	3000	7000	63,000
New EHR vendor	Cerner	Cerner	Epic	Epic
Prior EHR type	Homegrown	Best-of-breed*	Best-of-breed*	Homegrown

*Best-of-breed approach combines numerous different EHRs systems for different services and functions

Participants described several practices that influenced EHR transition success (Table 2). Practices occurred across different stages of implementation, including: (1) during planning and preparation (*pre-go-live*), (2) around the time of *go-live*, and (3) during sustainment and optimization of the new EHR (*post-go-live*). We describe practices according to their corresponding stage of implementation. Many of these practices, however, are relevant across the entire implementation process.

**Table 2:** Key EHR transition practices described by interview participants

Practices	Key components
Pre-go-live	
Communicate rationale and anticipated outcomes of the EHR change	• Emphasize the rationale for the change and address clinician concerns • Tailor communication to the specific experiences of the healthcare system • Set realistic expectations and address expected outcomes
Understand existing workflows	• Work with local stakeholders to understand existing workflows • Re-evaluate inefficiencies with current workflows • Use existing workflows as foundational knowledge for EHR changes
Plan for appropriate customization	• Align expectations with vendors early on and continuously revisit shared understanding about customization • Seek frontline clinician perspectives and input in meaningful ways • Identify engaged clinicians and provide protected time/support • Foster communication that limits inefficiencies and redundancies
Go-live	
Personalize training and support	• Tailor training to user needs • Engage local clinicians to contribute to training and support
Invest in robust internal support	• Identify influential and supportive superusers that can champion EHR transition efforts • Ensure adequate protected time and funding for local clinician training
Reduce workload expectations	• Find ways to mitigate additional work-related stressors • Decrease clinic capacity during go-live and tailor when to resume normal clinic load
Proactively address EHR transition challenges	• Listen to and engage frontline clinicians • Ensure transparent communication about challenges
Post-go-live	
Continue to invest in change	• Ongoing support and training following the first few weeks of the implementation are essential • Commit to continued system optimization • Communicate the approach, timing, and rationale for system optimization

### Pre-go-live

Participants conveyed that it is critical that leaders, end users, and vendors work together to support the change during the pre-go-live stage.

#### Communicate Rationale and Anticipated Outcomes of the EHR Change

Every system had unique baseline EHR configurations and motivation for change. In some, the older (legacy) EHRs were well-received, and the prospect of the transition was viewed with apprehension. In others, the older EHRs were perceived as unsustainable, which led to greater buy-in for the change. Communication about the change also differed. Opaque decision-making created animosity, while open communication acknowledging challenges was more well-received.

One participant criticized inadequate communication about the EHR transition, expressing a lack of understanding about the motivations for the change:

**I would have just loved to know why they did this.** Who picked it, why they picked it, and what do they think?... It's certainly not useful as a clinician for me, so there's got to be a reason it’s useful somewhere... I mean, at least then I would have some sort of sense why we picked such a bad program. [A02]

In contrast, a participant at another site readily articulated the rationale for the decision to transition between EHRs:

There was definitely some benefit to having a best of breed system, but there were too many systems being held together with strings…**and that’s why we eventually decided to move to a unified system…** [C07]

Another participant at the same site recounted that the robust communication from leadership, while at times bordering on excessive, was ultimately effective and well-received:

There was some information overload, I would say, with e-mails and the job aids and things. But, overall, they really did a good job. **So I would say perhaps they erred on the side of too much information**. [C11]

Indeed, a participant at another site advocated for extensive communication that includes granular information of anticipated changes:

I would even go so far as to say more communication at the cellular level. And what I mean by that is, when **you’re talking to the people who are actually on the ground level doing the work, and telling them the information from above** <…> **giving them specific details** about, ‘this <is> the time Epic is going to roll out, and this is exactly what we’re going to do, and your paperwork will be sitting at the desk at the nurse’s station, if you need it..’ [D04]

Some participants also felt that the pre-go-live communication must anticipate and honestly address the anxieties that non-clinicians may have about the impact of the EHR transition on their jobs:

we always met with the leadership of the non-clinicians and basically told them—**'Look, we’re getting a new system. People’s jobs are going to need to change. Nobody is going to lose their job because of that, but that being said, you need to make a change in the way you do your work;** otherwise, your job may not exist anymore.’ [D03]

#### Understand Existing Workflows

Many participants across systems highlighted the importance of developing a detailed understanding of the baseline state to better anticipate and plan for changes during the EHR transition. However, there was considerable variation in the approach to mapping existing workflows across sites and across services within a site.

One participant felt that inadequate understanding of existing workflows impaired the quality of the new EHR build.

… **you really have to have a very good understanding of your workflows** before you can make the build. And I think that we didn’t have a great understanding of workflows, so **a lot of time and energy were spent into making the build**, but then when you took the Epic product and **you applied kind of faulty workflows** or workflows that you didn’t fully… understand, it made it really hard to leverage the tool the way it really should be leveraged. [C10]

Underscoring this point further, a participant at another site described the benefit of leveraging multidisciplinary teams to understand existing workflows.

…no one knows the whole process; **everybody knows a little bit of their own piece of the process**, but no one can really tell you the history behind how this process developed, which has probably been iteratively developed over 20 years. So, **we had to really drill down** on that, hardcore. And, I think **because we did that, we ended up being very successful** in implementing this. [A06]

Participants also described a need to re-evaluate potential inefficiencies with the existing or legacy approach. One participant explained:

The other thing is that there’s a lot of **holdovers from legacy workflows**, and you don’t really gain the efficiency in the EHR. [C04]

#### Plan for Appropriate Customization

Participants described crucial decisions around how to customize the EHR to meet local needs. EHR customization was viewed as inevitable:

Even though <Epic> sell you something called a foundation system, which is allegedly their kind of out-of-the-box, you can’t use it as such. You really have to look at every parameter, decide what’s going to be on this drop down of choices, what’s this screen going to look like. So **there’s really a lot of customization and honing that has to be done**. [C02]

Although all systems required customization to some extent, highly customized systems took time to develop, were challenging to implement, and were difficult to maintain.

…if people raise the concern or <say>, ‘Hey, we need to … spend more time doing that’, there's always backpressure to say, ‘Hey, we need to get the system installed. We committed to install the system. We're not getting any benefit from the system until it is installed.’… So, **you're always trying to walk a fine line between improving the system or, if you will, doing better configuration and implementation versus timelines and actually get it installed**. [A03]

Customization was also seen as capable of creating challenges with established EHR functions.

…the vendor had a certain workflow that was laid out, <but> we had decided that **we wanted to mirror something that we had previously**, because we felt that that worked really well. Well, the trouble was, when you try to translate that into a <vendor> product, **a lot of times you broke a lot of things that were potentially to your advantage** that you lost because the vendor didn’t build it that way… we wanted to stick with Epic’s build as much as possible so that we wouldn’t disconnect some of the other tools that were in the back end of it. [D03]

Participants also reported that vendors often blamed system downtime and other deficits on customization:

What these [vendor] executives said is that since we chose to customize our [EHR] instead of getting—you know, the one ‘off the shelf,’ the ‘standard’ … we kind of hurt ourselves in that way. That **as long as we keep changing things and modifying them to what we want, [system down time] is going to keep happening**. [B03]

Recognizing these challenges, one participant recommended against customizing before go-live:

…my advice would be: **Don’t customize as much stuff as you can**. Like, take their basic package, work with it, and then customize afterwards. Versus customizing everything up front and then eventually trying to revert back to some of the typical features… I think that’s one of my biggest complaints of our go-live, is that **it was just so customized that even their help couldn’t help us all the time**. [D05]

For customization that is inevitable, participants universally recommended engaging local end users in decisions about the appropriate design.

I do think that one thing that’s really important is…**making sure that your build is stakeholder driven**. You don’t want IT people making decisions about how the system should work. **You want it to be the people who are actually going to be using the system,** who understand the implications of the decisions. [C01]

### Go-live

The period around go-live was incredibly challenging for many participants. Successful EHR transitions depended on leaders acknowledging these challenges and tailoring their approach to meet local needs.

#### Personalize Training and Support

Training was often the focus of frustration, with participants wishing for more individualized and interactive learning.

I just remember sitting in a big room in front of a computer, where they were like, ‘**Click here to do this, click here to do that**.’ You know, it wasn’t very useful. <…> Then they always have people walking around the room to see if everyone’s clicking on the same thing and it just becomes entirely pointless… And I can learn with an **individual trainer so much more usefully** on things that I would actually do. [A10]

[I would recommend] having more… supervised playground time where people could… work through workbooks with a trainer kind of walking people through those activities. So, **more interactive education as opposed to just a lecture**. [C10]

Participants alluded to trade-offs in using external (vendor-employed) vs. internal (trained superusers) personnel. External support was often perceived as insufficiently knowledgeable about local context.

…we learned a lot from [EHR transition at the first site], mostly what not to do. So we had relied on [vendor] training materials for our initial pilot site implementation, and that was the only time we used their material. So **from that point on, we created our own training material**… [A04]

#### Invest in Robust Internal Support

Internal superusers were generally perceived as better and more knowledgeable than external personnel. However, they often lacked structural support, including funding and protected time.

**The problem is a lot of it was unfunded**. It was not like people got more money to be a superuser. <…> So it’s a good concept, in theory, but it’s very hard to get people to be freed up to learn more about the application so that they can actually truly be helpful. [C02]

At one site, notably, the superusers *were* able to provide a robust amount of support because they were released from clinical duties and allowed to commit their entire workday to EHR support:

The first week we had 12 people or more in specifically outpatient urology that were superusers. And those **superusers did not have any patient load**, so we were like 0% productivity. We were just … teaching people that were seeing patients **how to navigate through the system, how to do it, how to bill, do their note more efficiently**. [D07]

One participant helped operationalize the process of developing robust internal support by describing their organization’s strategy of establishing multiple levels of internal training and support resources:

What [our organization and the EHR vendor] did was reach out, department-wide, across the enterprise, **looking for individuals that would be willing to step out of practice and work for the [EHR implementation], and do the direct department training of the [new] workflow** and things like that. So there was a lot of communication to staff as far as what the roles would be. They identified individuals that would want to first step out of the practice to be credential trainers, and then **also looked for individuals that wanted to stay within the practice and transition to what they would call a superuser.** Which would kind of help the rest of the members of the practice learn how to use [the EHR] most efficiently. [D08]

#### Reduce Workload Expectations

Participants recommended mitigating additional work stressors to account for the disruptive and time-consuming nature of learning a new EHR. All sites decreased clinical capacity during go-live, and this was universally appreciated:

They also seemed to have a pretty good awareness that **our clinic productivity was going to be down for quite some time**. So, you know, just… telling us that we expect this, it’s going to happen, and we’re ready for it, and we know it’s going to happen was kind of a good leadership message as well. [C05]

Another participant described the importance of having flexibility to continue to limit clinical capacity while still working to gain sufficient EHR proficiency:

If you came to us after the second week and said, ‘**We’re really struggling, we’ve got major issues**, things that need to be corrected,’ then they were still allowed to be reduced after that. So I think **there has to be some level of flexibility** to make sure that you’re meeting the needs of the groups that you’re serving. [D02]

#### Proactively Address EHR Transition Challenges

Leaders’ responses to EHR transition challenges differed with some being proactive and involved in identifying solutions while others more reluctant to address the problems.

One participant described frustration that their concerns with the system were not acknowledged by leadership:

We came to them very early on and said, ‘This is a problem. You need to go back and fix this program before you kill someone or endanger someone’s life. This should not be rolled out.’ And **initially, they were reluctant to listen to us**. …And **eventually, they listened to us, and they delayed**. [A09]

A contrasting perspective described leadership proactively acknowledging and communicating about emerging problems.

I think that was a **really positive piece of the leadership involvement... -- just being very present and…very transparent** about what went right and what went wrong… like, ‘Oh, we have this major problem. We didn’t realize this. Here's what we’ve done to fix it. Here's kind of the short-term fix.’ I think they did a really good job kind of sharing that and sharing problems that were occurring. [C10]

### Post-go-live

During the shift from go-live to post-go-live stages, EHR transition support tapers and the organizational momentum for the transition can slow down. Several participants noted that to ensure the best EHR transition outcomes, health systems need to embrace continuous improvement efforts.

#### Continue to Invest in Supporting Organizational Change

Sites varied in their approach to the post-go-live period. At some, insufficient resources were provided for ongoing support and training after vendor support withdrew.

One participant described the emotional toll that this change can have on end users:

…**after the initial wave of support leaves, we'd move on to the next implementation,** and they're kind of left like just to deal with everything. Now that everyone is gone, the reality sets in and the valley of despair, and people... get tired and frustrated and angry. [A04]

This stage was particularly important for learning the more intricate aspects of the new system.

The [classroom] phase [of training] is fine for teaching you how to turn it on, what the basic navigation functionality is and whatnot. **But you really need to use the system for a few weeks in actual care and then come back and then have a secondary training that focuses more on the optimization aspect of things**, because now you at least understand how it works. So then you can kind of build out how it would work for the way your workflow is. [D03]

This period also offered a valuable opportunity to refine and optimize the EHR:

Well after you implement you’ve got to **keep that driving force ensuring that users’ needs are met**. That’s an ongoing daily process. Don’t ever lose the momentum. If something doesn’t work, **go back to the drawing table** and get your team together and work it out. [C05]

## DISCUSSION

While EHR transitions are becoming increasingly common, there is limited evidence on practices that support these complex organizational changes. In this multi-site qualitative study of EHR transitions, we described clinician and staff perspectives on organizational practices perceived as most helpful. Transcending the context of individual sites and EHR systems, our findings may act as a roadmap for future EHR transitions. As VA and other systems strive to improve their ongoing EHR modernization efforts, our findings could offer guidance to national, regional, and local leaders to ensure the best possible EHR transition outcomes.

Several characteristics of this study are important and novel. First, in examining EHR-to-EHR transitions, we complement earlier work on paper-to-EHR transitions and highlight unique practices relevant for modern systems. There are key contextual differences that distinguish our findings from recommendations on initial paper-to-EHR implementations.^2–5^ The prior EHR context is especially relevant in the pre-go-live period when communicating about the change, assessing existing workflows, and planning for appropriate customization. Second, we expand limited prior EHR transition research by including multi-site qualitative data that offers detailed perspectives of EHR transition practices. Aligned with general recommendations from single-site studies and opinion pieces on EHR transitions,^9–12^ our study suggests specific practices and actions that leaders can take to support the organizational change. For example, to complement the common recommendation of engaging stakeholders,^9^ we presented participants’ perspectives on how relevant end users, leaders, and vendors can be engaged during the planning, implementation, and optimization of the new EHR. Additionally, our findings regarding the trade-offs involved in customizing an EHR system to reflect organization- or facility-level differences can inform the important but underexplored questions of how extensively to customize a system before implementation, and how to determine what can be standardized.^13,22^ Finally, we intentionally focused on end users’ experiences to collect their voice on effective organizational practices, and in doing so, we arm leaders with potential solutions to engage clinicians and staff in supporting EHR transition success.

Our findings are consistent with recent recommendations by Sittig et al. in their perspective piece, “Applying requisite imagination to safeguard electronic health record transitions.”^13^ The authors argue that EHR transitions require careful planning, collaboration, and imagination to proactively anticipate and avert harm, and they suggest that organizations use combination of technical expertise, strategic planning, and creative problem-solving. We concur with this proactive and collaborative approach to minimize the risk of errors and support end users through difficult EHR transitions, and we provide specific insights from end users on how to realize these goals.

In 2020, VA began an EHR transition of unprecedented size and scope, and it has already encountered numerous challenges. The Office of the Inspector General outlined several problems with the planning, implementation, and optimization of the new EHR,^15–18^ and many of these reports align with our findings. For example, EHR training was noted to be deficient at the initial site, with facility leaders describing the training as “button-ology,” and a local staff coordinator explaining, “it was just people sitting down and learning to use the buttons and not having any context for what they were doing.”^18^ We have communicated with VA leadership about our findings. This work could act as a roadmap for new strategies and course corrections to optimize VA’s EHR modernization efforts. As VA strives to make these improvements, it has a powerful opportunity to identify and test strategies that support more successful EHR transitions.

This study has potential limitations. Our findings may not transfer to other individuals or settings. Notably, we included few health system leaders, and we cannot comment on their unique perspectives of effective change strategies. EHR vendors have unique insights on conducting these transitions, although we were unsuccessful in engaging vendors in interviews. We included few many non-clinical roles and cannot comment on perspectives of local informatics and information technology staff. We only selected sites that transitioned to one of two major EHR systems, although none of the practices that we identified seemed to be specific to the selected EHR. Finally, the purpose of this qualitative study was to identify practices that transcend site- and EHR-specific context, and limitations in scope and word limit preclude us from offering a detailed analysis of the unique experiences of each study site. We plan to address this limitation in future research.

In conclusion, this multi-site study of lessons learned from EHR transitions identified specific organizational practices that can act as a roadmap across EHR implementation stages. As more health systems look to new EHRs to address pressing healthcare challenges, our findings may provide a valuable resource for leaders who must navigate these complex changes.

### Supplementary Information

Below is the link to the electronic supplementary material.

Supplementary file1 (DOCX 28 kb)
