# Supplementary material for: Practices Supporting Electronic Health Record Transitions: Lessons from Four US Healthcare Systems
Source: J Gen Intern Med. 2023 Oct 5;38(Suppl 4):1015–22. doi: 10.1007/s11606-023-08279-0 (PMC10593676; doi:10.1007/s11606-023-08279-0)
Supplement: Supplementary file 1 — Supplementary file1 (DOCX 28 kb) [file 11606_2023_8279_MOESM1_ESM.docx]

**interview guide**

**I. Implementation Process**

1. Could you give me a general **overview** of when and how the Cerner / EPIC implementation happened in your organization?
   1. How did you first learn about your organization’s decision to adopt Cerner / EPIC? Do you remember how you felt / what you thought at that moment? What about your colleagues?
   2. In what way were you involved in the implementation?
2. Please think back to the time period before the go-live. To your knowledge, what kind of **preparations** were taking place during that time? *(Probe about needs assessment, workflow mapping, workgroups/meetings, etc.*)
   1. If the interviewee was involved in preparation as a champion/leader: How were you involved at this stage? Who else was involved in this work? Tell me about some challenges involved. What were the consequences of this work? What do you think was done well? What would you have done differently, if you had a chance?
   2. If the interviewee was a frontline clinician: What do you think was done well with these preparations? What would you have done differently, if you had a chance?
3. What do you remember about **education or training** that was available for clinicians before and during the go-live?
   1. What kind of **training** related to Cerner / EPIC, if any, have you personally completed before or during the go-live?
   2. What worked well about the training you received, and why?
   3. What didn’t work well, and why?
   4. Did the training make you feel more comfortable with Cerner / EPIC? Why or why not?
   5. What other sorts of training or education were available, as far as you remember?
4. **Champions**:
   1. If a self-described champion: Can you describe what being a champion involved? What about your work as a champion that you think you did well? What would you change? What about other champions – what sorts of things did they do well? What would you change?
   2. If not a champion: Tell me about local champions involved in the implementation. As far as you remember, what sorts of tasks or roles did the champions have? What was it that the champions did well? What would you change?
5. **Help/support**:
   1. If a front-line provider: During the initial Cerner / EPIC roll out, if you had a problem with the system or a question about it, what did you do? Who would you turn to for **help** or **support**? *(Ask about Cerner personnel, local IT, champions, other colleagues.)*
   2. If a leader: During the initial Cerner / EPIC roll out, if clinicians had a problem with the system or a question about it, what did they do? Who would they turn to for help or support? (*Ask about Cerner personnel, local IT, champions, other colleagues.)*
   3. Both: What are some things that you wish had been done differently about the help or support available during the implementation? What are some things that worked well?
6. What was the role of the **leadership** during the initial Cerner / EPIC rollout?
   1. If had a leadership role: As a leader, could you describe your involvement in the Cerner / EPIC rollout? What do you think you did well? What do you wish you had done differently?
   2. Thinking about your **direct leadership**, what was their involvement? Was there anything about the leadership involvement that worked well? Something you wish had been done differently?
   3. Thinking about the **senior leadership** (above your direct leadership), in what way were they involved? Was there anything about the leadership involvement that worked well? Something you wish had been done differently?
7. Now I will ask about the effects of Cerner / EPIC implementation on your **work life.** Thinking back to the time period around the Cerner / EPIC roll-out, how did it affect your ability to do your job?
   1. Working hours and efficiency
   2. Your ability to provide high-quality and safe care
   3. Your interactions with patients
   4. Your relationships with colleagues
8. **Work satisfaction**. Tell me about how the Cerner / EPIC roll-out affected the way you felt about your job, if at all.
   1. Did you feel content, burnt out, stressed, etc.?
9. I am also interested in how, in your perception, your **organization** was affected by the transition to Cerner / EPIC. Thinking back to the Cerner / EPIC roll-out, what can you tell me about the impact of the roll-out on:
   1. Patient care quality / safety (including ability to track quality indicators)
   2. Staff satisfaction and turnover
10. Thinking about Cerner / EPIC implementation as a whole, what lessons do you think other organizations, including the VA, learn from yours?

**II. Current Proficiency & Well-Being**

1. Generally speaking, what does **being proficient** in an EHR mean to you?
2. How **proficient** are you yourself with Cerner / EPIC now? Can you give an example (illustrating your proficiency / lack of proficiency)?
   1. Do you think there is a relationship, one way or another, between being proficient in an EHR and providing high-quality care? Please elaborate.
3. Are you **continuing to learn** about Cerner / EPIC and its applications? In what ways, if so? (Probe about online courses, videos, in-person education, peer leaders, consultants)
   1. [If self-described as proficient] Do you have any recommendations on how other clinicians can become more adept in the use of Cerner / EPIC?
4. I also wanted to ask about your thoughts or feelings related to **burnout**. First of all, what does burnout mean to you?
5. Do you **feel burnt** out from your work? Please elaborate or give an example.

**III. Post-Implementation**

1. Since the initial rollout of Cerner / EPIC, what kind of **support** has been available at your organization (*local IT, Cerner / EPIC tech support, scribes*)?
2. What else should we know about what it was like in your **organization** once the implementation of Cerner / EPIC was completed?
   1. Work routines / workflow
   2. Care quality / safety
   3. Relationships between colleagues
   4. Staff satisfaction and turnover
3. Please tell me about the impact of the COVID-19 situation on your organization.
   1. Could you talk about changes in workflows or routines that you’ve observed?
   2. Could you tell me how this has impacted your own and your colleagues well-being?

**IV. Conclusion**

1. Is there anything else you want us to know or anything else you’d like to add?
